# Anti-Inflammatory and Antinociceptive Effects of Salbutamol on Acute and Chronic Models of Inflammation in Rats: Involvement of an Antioxidant Mechanism

**DOI:** 10.1155/2012/438912

**Published:** 2012-05-14

**Authors:** Hulya Uzkeser, Elif Cadirci, Zekai Halici, Fehmi Odabasoglu, Beyzagul Polat, Tugba Nurcan Yuksel, Seda Ozaltin, Fadime Atalay

**Affiliations:** ^1^Department of Physical Medicine and Rehabilitation, Erzurum Regional Training and Research Hospital, 25240 Erzurum, Turkey; ^2^Department of Pharmacology, Faculty of Pharmacy, Atatürk University, 25240 Erzurum, Turkey; ^3^Department of Pharmacology, Faculty of Medicine, Atatürk University, 25240 Erzurum, Turkey; ^4^Department of Biochemistry, Faculty of Pharmacy, Atatürk University, 25240 Erzurum, Turkey

## Abstract

The possible role of **β**-2 adrenergic receptors in modulation of inflammatory and nociceptive conditions suggests that the **β**-2 adrenergic receptor agonist, salbutamol, may have beneficial anti-inflammatory and analgesic effects. Therefore, in this study, we induced inflammatory and nociceptive responses with carrageenan-induced paw edema or cotton-pellet-induced granuloma models, both of which result in oxidative stress. We hypothesized that salbutamol would prevent inflammatory and nociceptive responses by stimulating **β**-2 adrenergic receptors and the prevention of generation of ROS during the acute inflammation process in rats. Both doses of salbutamol used in the study (1 and 2 mg/kg) effectively blocked the acute inflammation and inflammatory nociception induced by carrageenan. In the cotton-pellet-induced granuloma test, both doses of salbutamol also significantly decreased the weight of granuloma tissue on the cotton pellets when compared to the control. Anti-inflammatory and analgesic effects of salbutamol were found to be comparable with those of indomethacin. Salbutamol decreased myeloperoxidase (MPO) activity and lipid peroxidation (LPO) level and increased the activity of superoxide dismutase (SOD) and level of glutathione (GSH) during the acute phase of inflammation. In conclusion, salbutamol can decrease acute and chronic inflammation, possibly through the stimulation of **β**-2 adrenergic receptors. This anti-inflammatory effect may be of significance in asthma treatment, where inflammation also takes part in the etiopathology. This study reveals that salbutamol has significant antioxidative effects, which at least partially explain its anti-inflammatory capabilities. These findings presented here may also shed light on the roles of **β**-2 adrenergic receptors in inflammatory and hyperalgesic conditions.

## 1. Introduction

Inflammatory diseases such as rheumatoid arthritis, hepatitis, and asthma are major causes of morbidity in humans. Chronic inflammation is now well known to also lead to the development of cancer [1], cardiovascular diseases [2], and neurodegenerative diseases [3]. In inflammatory diseases, the most common complaint of the patient is accompanying nociception and fever. Although nonsteroidal anti-inflammatory drugs (NSAIDs), especially indomethacin, are the drugs of choice in the treatment of inflammatory diseases [4] and are highly effective, they have a number of deleterious side effects, such as gastrointestinal ulcers and even bleeding [4, 5]. So investigators are still looking for new analgesic drugs with fewer side effects for the treatment of inflammation and nociception.

The most common mechanism for the anti-inflammatory and analgesic effects of NSAIDs is via the inhibition of prostaglandin synthesis by the COX enzyme [6]. On the other hand, some other mechanisms such as the L-arginine/nitric oxide pathway or the serotonergic system have also been suggested for analgesic effects of NSAIDs [7, 8]. In addition, Lizarraga and Chambers claim a role for the opioidergic system in the analgesic effect mechanism of NSAIDs [9]. However, recent studies now claim a role for *β*-2 adrenergic receptors in the anti-inflammatory and analgesic effects of NSAIDs [10, 11]. A role for adrenergic *β*-2 receptors has been previously demonstrated in inflammatory conditions [12, 13]. While there are some publications that indicate that activation of *β*-2 adrenergic receptors may be involved in the increased nociceptive sensitivity [14], and inflammatory hyperalgesia [15] recent studies suggested that *β*-2 adrenergic receptor activation can inhibit nociception and inflammation [16–18].

 Recent literature on a possible role of *β*-2 adrenergic receptors in modulation of inflammatory and nociceptive conditions led us to hypothesize that *β*-2 adrenergic receptor agonists, such as salbutamol, may provide anti-inflammatory and analgesic relief for nociceptive inflammatory conditions. Salbutamol is a well-known drug that is commonly used in the treatment of bronchial asthma [19]. Salbutamol selectively binds to and activates *β*-2 adrenergic receptors on the surface of many cells. The inhibitory effect on inflammatory processes is seen primarily for CD4 cells but also for other leucocytes with a high density of *β*-2 receptors such as monocytes, macrophages, and Langerhans cells [20–22]. Also anti-inflammatory effects of *β*-2 adrenergic receptors on pulmonary inflammation models [23] support the role of *β*-2 adrenergic receptors in inflammatory conditions.

 The binding of the *β*-2 receptor agonist to these cells inhibits activation of the expression of inflammatory genes and thereby their proinflammatory cytokines, such as interleukin-2 and interferon-*γ*. Salbutamol also inhibits superoxide generation and peroxidase release from stimulated human granulocytes [24]. These effects can be investigated for their therapeutic potential using inflammation models. For example, one hour after subcutaneous injection of carrageenan into the rat paws nociception model or inflammation model, in which vascular permeability increases and leukocyte migration occurs, involves inflammatory mediators including neutrophil-derived active oxygen species and free radicals, such as hydrogen peroxide, superoxide and the hydroxyl radical [25–27] nitric oxide, prostaglandins, and cytokines [28]. Also, neutrophil accumulation liberated proinflammatory mediators such as cytokines, including TNF-*α* and IL-1*β*, are considered to be proinflammatory agents that stimulate the cellular chemotaxis and serve to further increase tissue inflammation [29]. However, no studies have yet investigated the anti-inflammatory and analgesic potential of salbutamol in relation to these oxidative parameters.

Therefore, in the present study, we induced inflammatory and nociceptive responses with carrageenan-induced paw edema and with cotton-pellet-induced granuloma models. Both of these treatments result in oxidative stress, and we hypothesized that salbutamol would prevent inflammatory and nociceptive responses by stimulating *β*-2 adrenergic receptors and reducing the generation of reactive oxygen species (ROS) during acute inflammation process in rats. 

## 2. Materials and Methods

### 2.1. Animals

In this study, we used a total of 90 male Albino Wistar rats obtained from the Medical Experimental Research Centre, Atatürk University (ATADEM). The animals weighed between 200 and 220 g and were fed under normal temperature conditions (22°C) in separate groups before the experiments. Animal experiments were performed in accordance with the national guidelines for the use and care of laboratory animals, and the study was approved by the local animal care committee of Atatürk University.

### 2.2. Chemicals

All chemicals for laboratory experimentation, including carrageenan, were purchased from Sigma Chemical (Germany). Thiopental sodium was purchased from IE Ulagay A. S. Istanbul, Turkey; indomethacin was purchased from Deva, Turkey; salbutamol was purchased from GlaxoSmithKline, and propranolol was purchased from Sanofi Aventis, Istanbul, Turkey.

### 2.3. Carrageenan-Induced Paw Edema in Rats

In the first series of experiments, the anti-inflammatory effects of salbutamol and indomethacin on carrageenan-induced paw edema were studied in rats [30]. The rats were divided into 4 groups (*n* = 6) for experimental procedure. Three rat groups received salbutamol 1 mg/kg, salbutamol 2 mg/kg, or indomethacin 25 mg/kg by oral gavage. Rat doses of salbutamol differ from 3 *μ*g to 60 mg/kg [31, 32]. In this study we selected 1 and 2 mg/kg doses of salbutamol [33, 34]. In acute inflammation model and hyperalgesia model we studied indomethacin at 25 mg/kg dose, which has been previously used [35–37]. The reason why we used the 25 mg/kg dose in acute experiments is that we aimed to compare the effects of salbutamol with the highest dose of the reference drug. All drugs were suspended in distilled water as vehicle. So the control group (4th group) received an equal volume of distilled water as vehicle. One hour after drug administration, 0.1 mL of 1% carrageenan was injected into the hind paw of each rat in each group. Before the carrageenan injection, the normal paw volumes of the rats were measured with a plethysmometer. The carrageenan-induced increase in the paw volume was measured four times at one-hour intervals. Namely, the paw volumes were measured for every 60 minutes times four hours after carrageenan injection [38–40]. The effects of the drugs were determined by comparing the results of the drug-treated groups with those of the control group. At the end of the experiment, paw tissues of all animals, as well as from an additional untreated group of healthy animals, were collected for biochemical examination. All of the paw tissues were immediately transferred to −80°C.

In the second series of experiments we investigated whether anti-inflammatory activity of salbutamol is related to *β*-2 adrenergic receptor stimulation or not. For this purpose a total of 12 rats were divided into 2 groups (*n* = 6). The first rat group received 40 mg/kg dose of propranolol, which was suspended in distilled water by oral gavage. One hour after propranolol administration the rat group received 2 mg/kg dose of salbutamol by oral gavage. The control group (2nd group) received an equal volume of distilled water as vehicle, and anti-inflammatory activities were determined as described above.

### 2.4. Carrageenan-Induced Inflammatory Paw Hyperalgesia in Rats

In this series of experiments, the analgesic effects of salbutamol and indomethacin on carrageenan-induced inflammatory paw hyperalgesia were studied in intact rats [41]. The rats were divided into 4 groups (*n* = 6). Drug administration and carrageenan treatment were repeated exactly as described in Section 2.3. Prior to carrageenan injection, the normal nociceptive thresholds of the rats were measured with a Basile algesimeter that measures mechanical reflex threshold. Carrageenan-induced decrease in the nociceptive threshold was measured three times at one-hour intervals. Namely, the nociceptive thresholds were measured for every 60 minutes times three times after carrageenan injection. The analgesic effects of the drugs were determined by comparing the results of the drug-treated groups with those of the control group.

### 2.5. Cotton Pellet Granuloma Test

In this part of experiment, we used 24 rats divided into 4 groups to examine the effects of salbutamol and indomethacin on the proliferative phase of inflammation [42]. For this purpose we used the cotton pellet test, which is a chronic inflammation model used for evaluating the antiproliferative effects of drugs [42, 43]. In this model, a short time after the initiation of acute inflammation, proliferative cells developed and inflammation became chronic. Monocyte-macrophages infiltration and fibroblast proliferation occur in chronic inflammation [44]. Also in the cotton-pellet-induced chronic inflammation model, cotton pellet, which we applied in interscapular area, induced a chronic inflammation process. In this process monocyte migration, liquid accumulation, apoptosis, damage and so on will occur in the surrounding tissue of the pellets and these accumulations will produce a granulation tissue that covers the pellets. Salbutamol at 1 mg/kg and 2 mg/kg doses was administered to the first two groups of rats, and 5 mg/kg of indomethacin [45] was given orally with the aid of gavages to a third group. The reason why we used 5 mg/kg dose of indomethacin in chronic administration is that high dose (25 mg/kg) indomethacin has quite harmful effects on stomach resulting in stomach bleeding and even death in chronic administrations. All drugs were suspended in distilled water as vehicle. The control group received an equal volume of distilled water. Thirty minutes after the administration of drugs, rats were anesthetized with 20 mg/kg of thiopental sodium. Cotton pellets, weighing 7 ± 1 mg and prepared under sterile conditions, were then implanted subcutaneously (sc) in the interscapular area. Drugs were administered once a day for a period of 7 days. On the 8th day, rats were euthanized with a high-dose (50 mg/kg) of thiopental sodium. Cotton pellets with the granuloma tissue that involves migrated monocytes, accumulated liquid, and fibroblasts were removed and weighed. Effects of the drugs on chronic inflammation were determined by comparing the results obtained for the test groups with the results of the control group.

### 2.6. Biochemical Estimations

After the macroscopic analyses, superoxide dismutase (SOD) and myeloperoxidase (MPO) enzyme activities and the glutathione (GSH) and lipid peroxidation (LPO) levels in rat paw tissues were determined. To prepare the tissue homogenates, whole paw tissues were ground with liquid nitrogen in a mortar. The ground tissues (0.5 g each) were then treated with 4.5 mL of appropriate buffer. The mixtures were homogenized on ice using an ultraturrax homogenizer (IKA-Germany) for 15 min. Homogenates were filtered and centrifuged by using a refrigerated centrifuge at 4°C. Then, these supernatants were used for determination of the enzymatic activities. All assays were carried out at room temperature in triplicate.

#### 2.6.1. Superoxide Dismutase Activity

As outlined by Sun et al. [46] superoxide dismutase estimation was based on the generation of superoxide radicals produced by xanthine and xanthine oxidase, which react with nitro blue tetrazolium to form formazan dye. Superoxide dismutase activity was then measured at 560 nm as the degree of inhibition of this reaction and was expressed as millimoles per minute per milligram tissue (mmol/min/mg tissue).

#### 2.6.2. Myeloperoxidase Activity

Myeloperoxidase activity was measured according to a modified method of Bradley et al. [47]. The homogenized samples were frozen and thawed three times and centrifuged at 1500 g for 10 min at 4°C. Myeloperoxidase activity in the supernatant was determined by adding 100 mL of the supernatant to 1.9 mL of 10 mmol/L phosphate buffer (pH 6.0) and 1 mL of 1.5 mmol/L o-dianisidine hydrochloride containing 0.0005% (wt/vol) hydrogen peroxide. The changes in absorbance at 450 nm for each sample were recorded on a UV-Vis spectrophotometer. Myeloperoxidase activity in tissues was expressed as micromoles per minute per milligram tissue (*μ*mol/min/mg tissue).

#### 2.6.3. Total Glutathione (GSH) Determination

The amount of GSH in the paw tissues was measured according to the method of Sedlak and Lindsay [48]. The paw tissue homogenized in 2 mL of 50 mM Tris-HCl buffer containing 20 mM EDTA and 0.2 M sucrose, pH 7.5. The homogenate was centrifuged at 4200 rpm for 40 min at 4°C, and then the supernatant was used to determine GSH using 5,5-dithiobis(2-nitrobenzoic acid). Absorbance was measured at 412 nm using a spectrophotometer. The results of the GSH level in the rat paw tissues were expressed as nanomoles per milligram tissue (nmol/mg tissue).

#### 2.6.4. Determination of Lipid Peroxidation Level

Lipid peroxidation levels in paw tissues were determined by estimating malondialdehyde (MDA) using the thiobarbituric acid test [49]. Briefly, the paw tissues were promptly excised and rinsed with cold saline. To minimize the possibility of interference of hemoglobin with free radicals, any adhering blood or bristles on the epidermis were carefully removed. The paw tissues were weighed and homogenized in 10 mL of 100 g/L KCl. The homogenate (0.5 mL) was added to a solution containing 0.2 mL of 80 g/L sodium lauryl sulfate, 1.5 mL of 200 g/L acetic acid, and 1.5 mL of 8 g/L 2-thiobarbiturate and 0.3 mL distilled water. The mixture was incubated at 98°C for 1 h. Upon cooling, 5 mL of n-butanol/pyridine (15 : 1) was added. The mixture was vortexed for 1 min and centrifuged for 30 min at 4000 rpm. The supernatant absorbance was measured at 532 nm. A standard curve was generated using 1,1,3,3-tetramethoxypropane. All samples were measured in triplicate. The results were expressed as nmol MDA per gram wet tissue (nmol/g tissue).

### 2.7. Statistical Analyses

 Data for acute and chronic inflammation models and acute nociceptive thresholds model were subjected to one-way analysis of variance (ANOVA) using SPSS 13.0 software. Only the data for propranolol examination were subjected to “two-independent-sample *t*-test.” Differences among the groups were obtained using the LSD option and were considered significant at *P* < 0.05. A statistical analysis of oxidative enzymes was carried out using one-way ANOVA followed by Duncan's multiple range test (DMRT) using the SPSS software package, version 13.00, and were considered significant at *P* < 0.05. All the results were expressed as mean ± SE.

## 3. Results

### 3.1. Carrageenan-Induced Paw Edema in Rats

As seen in Table 1, both doses of salbutamol and indomethacin significantly decreased carrageenan-induced paw edema formation in rats. The anti-inflammatory effects of 1 mg/kg dose of salbutamol were determined as 43.9%, 43.4%, 44%, and 37.5%, respectively, for the 1st, 2nd, 3rd, and 4th hours. For the same hours, a 2 mg/kg dose of salbutamol produced 44.6%, 48.1%, 48.5%, and 48.8% anti-inflammatory effects, respectively. In comparison, the anti-inflammatory effects of indomethacin were 27.7%, 40.3%, 42.7%, and 48.4%, respectively, for the same time intervals.

**Figure 1:**
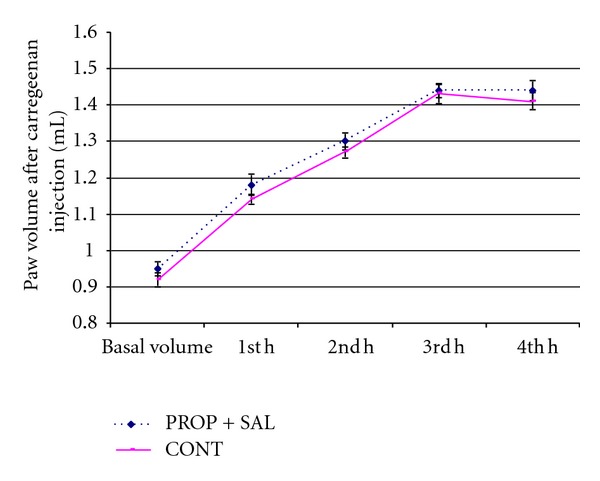
Effects of combination of salbutamol (2 mg/kg) and propranolol (40 mg/kg) (PROP + SAL) on carrageenan-induced inflammatory paw volume in rats. CONT: control.

In the second series of our experiments we determined that 40 mg/kg dose of propranolol reversed the anti-inflammatory effect of salbutamol (2 mg/kg). Namely, salbutamol could not inhibit inflammation formation when *β*-adrenergic receptors were blocked (Figure 1).

### 3.2. Carrageenan-Induced Paw-Hyperalgesia in Rats

As seen in Table 2, both doses of salbutamol and indomethacin significantly prevented the carrageenan-induced decrease in nociceptive thresholds in rat paws till the 3rd hour of carrageenan injection. In the 1st hour after carrageenan injection, the 1 and 2 mg doses of salbutamol and 25 mg/kg dose of indomethacin produced 57%, 61.1%, and 71.6% analgesic effects, respectively. The same doses of the drugs produced 44.2%, 47.8%, and 55% analgesic effects in the 2nd hour. In the 3rd hour the analgesic effects of salbutamol were lower than those in 1st and 2nd hours (36.9% for 1 mg/kg dose and 42.9% for 2 mg/kg dose). In the 3rd hour, indomethacin produced a 58.5% analgesic effect.

### 3.3. Cotton Pellet Test in Rats

On the 8th day, mean weights of moist pellets removed from the rat groups administered salbutamol (1 and 2 mg/kg) and indomethacin (5 mg/kg) and the control group were 94.0 ± 4.1 mg, 116.7 ± 3.0 mg, 41.3 ± 3.4 mg, and 168.7 ± 4.3 mg, respectively. According to these results, the effects of salbutamol 1 mg/kg, salbutamol 2 mg/kg, and indomethacin on chronic inflammation were evaluated as 44.3%, 30.8%, and 75.5%, respectively (Table 3).

### 3.4. Biochemical Analyses

**Figure 2:**
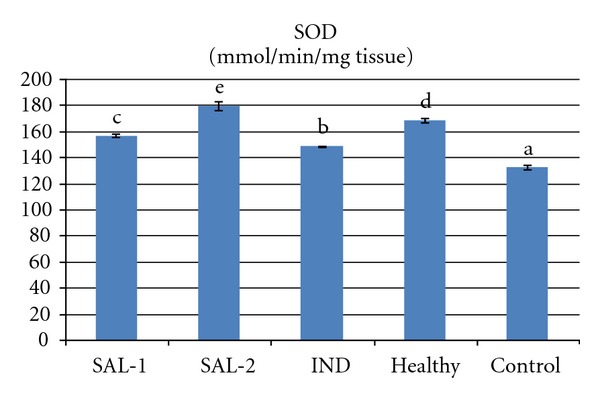
Effects of salbutamol (SAL) and indomethacin (IND) on superoxide dismutase (SOD) activity in carrageenan-injected paw tissues. Means in the same column by the same letter are not significantly different and the means in the same column by different letters demonstrate significant differences between the groups according to the Duncan test (*α* = 0.05). In the above figure, all columns have different letters. This demonstrates that values in these columns are statistically different from each other.

**Figure 3:**
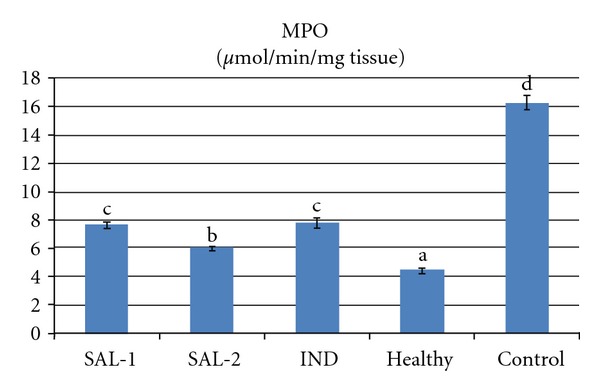
Effects of salbutamol (SAL) and indomethacin (IND) on myeloperoxidase (MPO) activity in carrageenan-injected paw tissues. Means in the same column by the same letter are not significantly different and the means in the same column by different letters demonstrate significant differences between the groups according to the Duncan test (*α* = 0.05). In the above figure, the letter for the means in SAL-1 and IND columns is the same: “c.” This demonstrates that values in these lines are not statistically different from each other. However, the lines with the letter “c” are statistically significant from the lines with the letters “a,” “b,” and “d.”

**Figure 4:**
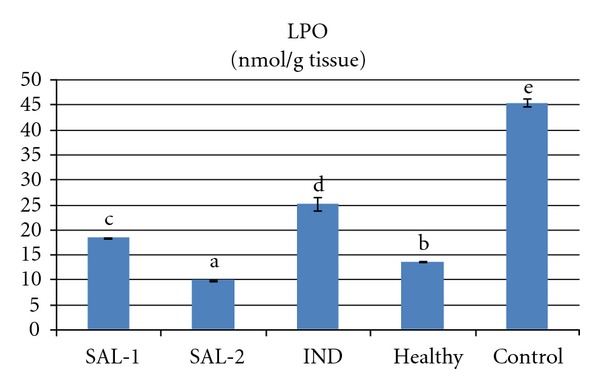
Effects of salbutamol (SAL) and indomethacin (IND) on lipid peroxidation (LPO) level in carrageenan-injected paw tissues. Means in the same column by the same letter are not significantly different and the means in the same column by different letters demonstrates significant differences between the groups according to the Duncan test (*α* = 0.05). In the above figure, all columns have different letters. This demonstrates that values in these columns are statistically different from each other.

**Figure 5:**
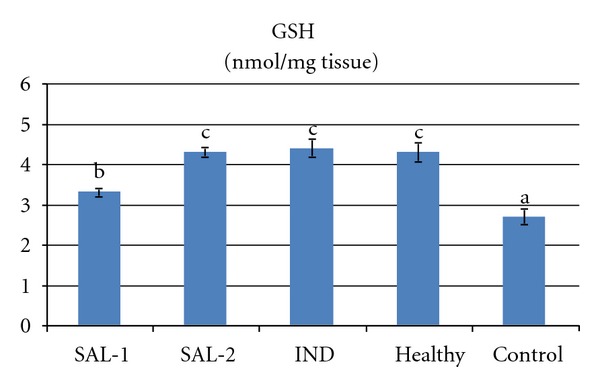
Effects of salbutamol (SAL) and indometacin (IND) on reduced glutathione (GSH) level in carrageenan-injected-paw tissues. Means in the same column by the same letter are not significantly different and the means in the same column by different letters demonstrate significant differences between the groups according to the Duncan test (*α* = 0.05). In the above figure, the letter for the means in SAL-2, IND, and healthy columns is the same: “c.” This demonstrates that values in these lines are not statistically different from each other. However, the lines with the letter “c” are statistically significant from the lines with the letters “a” and “b.”

Carrageenan injection to rat paws produced a significant increase in MPO activity and LPO level. However both doses of salbutamol significantly prevented the carrageenan induced increase in theses parameters (*P* < 0.05). Indomethacin administration also significantly decreased (*P* < 0.05) the MPO activity and LPO level when compared to control group that received carrageenan alone. The 2 mg/kg dose of salbutamol was more effective in decreasing MPO activity and LPO level than the 1 mg/kg dose of salbutamol or the 25 mg/kg dose of indomethacin (Figures 3 and 4). Carrageenan treatment resulted in a significant decrease in the activity of SOD and level of GSH, which were increased by salbutamol and indomethacin administration (*P* < 0.05). The 2 mg/kg dose of salbutamol was the best of the three drug treatments in terms of increasing SOD activity and GSH level (Figures 2, 3, 4, and 5).

## 4. Discussion

This study investigated the protective effect of salbutamol, a *β*-2 adrenergic receptor agonist drug used in bronchial asthma, on acute (carrageenan-induced) and chronic (cotton pellet induced) inflammation models and on a carrageenan-induced nociception model. Tissue levels and activities of LPO, GSH, MPO, and SOD were used to estimate antioxidant effects.

 Our study demonstrated that both doses of salbutamol (1 and 2 mg/kg) effectively reduced the acute inflammation and inflammatory nociception associated with carrageenan injection. Carrageenan application is known to produce an inflammatory response that peaks at three hours, resulting in hyperalgesia [50]. In this experimental inflammation model, levels of inflammatory mediators have been reported to increase fourfold between the 1st and 3rd hours after a carrageenan injection and then to remain high for several hours thereafter [51]. In the present study, the preventive effect of salbutamol on inflammation and related nociception formation was comparable with that of indomethacin, a potent anti-inflammatory drug. Both salbutamol and indomethacin exerted anti-inflammatory and analgesic effects; however, it is known that indomethacin has quite harmful effects on stomach tissue such as ulcer, perforation, and even bleeding. The ratio of occurrence for the side effects is approximately 35–50%, and as a result of present side effects 20% of patients are forced to stop indomethacin therapy [52]. Long-acting beta agonists have also side effects in high doses on cardiovascular system by activating sympathetic system. However, these side effects are not severe in salbutamol usage because it has a short half-life [53]. So salbutamol may be safer than indomethacin in inflammation treatment.

 Salbutamol selectively activates the *β*-2 adrenergic receptors and is clinically used for treatment of acute and chronic asthma [19]. In our previous studies, we suggested that *β*-2 adrenergic receptors may play a role in the suppression of inflammation as stimulation of these receptors would produce anti-inflammatory and, consequently, analgesic effects [10, 11]. There is also some evidence that catecholamines suppress immune cell functions in inflammatory tissues and that they produce this suppressive effect via activation of *β*-adrenergic receptors [12]. In another study, adrenergic agents were shown to suppress the immune response (the production of TNF-*α*) via the direct stimulation of *β*-adrenergic receptors on inflammatory immune cells [13]. Also some recent studies suggested that *β*-2 adrenergic receptor activation can inhibit nociception and inflammation [16–18]. These previous reports concerning the roles of *β*-2 adrenergic receptors in inflammatory conditions support a preventive role for salbutamol in inflammatory and related hyperalgesic conditions, as postulated in the present study.

 The effects of salbutamol and indomethacin on chronic phases of inflammation in the cotton pellet granuloma test of intact rats were also supportive of this hypothesis. The cotton pellet test is a chronic inflammation model used for evaluating the antiproliferative effects of drugs [43]. Both doses of salbutamol significantly decreased the weight of cotton pellets when compared to the control. A short time after the initiation of acute inflammation, proliferative cells developed and inflammation became chronic. Prevention of collagen fiber formation and suppression of mucopolysaccharides are indicators of the antiproliferative effect of anti-inflammatory agents [54]. Monocyte infiltration and fibroblast proliferation occur in chronic inflammation instead of neutrophil infiltration and exudation [44]. Activated monocyte-macrophages are blood cells that have antitumor and antimicrobial functions in addition to phagocytotic functions against pathogens [55]. Salbutamol selectively binds to and activates *β*-2-adrenoceptors, which are molecules on the surface of many cells, such as CD4 cells, leukocytes, monocytes, macrophages, and Langerhans cells [20–22]. The binding of a *β*-2 receptor agonist to these cells results in stimulation of the receptor and inhibition of expression of inflammatory genes. This prevents the production of proinflammatory cytokines, such as interleukin-2 and interferon-*γ* [24, 56], and effectively suppresses inflammation. Therefore, the primary effects of salbutamol on the chronic phase of inflammation may be associated with its effects on the *β*-2 adrenergic receptors located on the monocytes and macrophages that comprise the basic components of chronic inflammation. In contrast to our hypothesis Oliveira et al. and Pelegrini-da-Silva et al. suggested that serotonin-induced inflammatory hyperalgesia and temporomandibular joint inflammation are mediated by sympathetic amines-dependent mechanism that involves the activation of peripheral *β*-2 adrenergic receptors [15, 57]. However, expression of *β*-2 adrenergic receptors within the nociceptive system suggested their potential implication in nociception and pain and studies suggesting that *β*-2 adrenergic receptor agonists may potentially offer an alternative therapy to antidepressant drugs for the chronic treatment of neuropathic pain [17, 18, 58]. These data bring up such a dilemma: “Are central *β*2 adrenergic receptors involved in the analgesic effects of salbutamol or is it only consequence of the reduction in the inflammatory process?”. In this point, previous studies which claimed that surgical stress induces sympathetically activated release of endogenous opioids from inflammatory cells and subsequent analgesia via activation of peripheral opioid receptors [59] make us hypothesize that salbutamol shows its antihyperalgesic effects by inhibition of inflammation and peripheral sympathetic activation. Also in our study propranolol, a *β*-adrenergic receptor antagonist, administration reversed the anti-inflammatory effects of salbutamol suggesting that salbutamol mediated its anti-inflammatory effects via *β*-2 adrenergic receptors. However, future studies comparing effects of both peripheral and central *β*-2 adrenergic receptors are required for a better understanding.

 An acute inflammatory process is comprised of inflammation mediators including neutrophil-derived ROS, nitric oxide [60, 61] prostaglandins, and cytokines [62]. Also neutrophil accumulation liberated proinflammatory mediators such as cytokines, including TNF-*α* and IL-1*β*, are considered to be proinflammatory agents that stimulate the cellular chemotaxis and serve to further increase tissue inflammation [29]. ROS play an important role in the pathogenesis of many diseases, such as rheumatoid arthritis, local or systemic inflammatory disorders, ischemia-reperfusion injury, atherosclerosis, cancer, and respiratory distress syndrome [61, 63–65]. In respiratory diseases such as asthma, selective stimulation of *β*-2 adrenergic receptors results in NO production [66], which suggests that salbutamol produces its bronchodilator effects by stimulating *β*-2 adrenergic receptors, resulting in activated NO production [67–69]. Salbutamol also inhibits superoxide generation and peroxidase release from stimulated human granulocytes [24]. However, the effects of salbutamol on other parameters related to oxidative stress in inflammatory conditions have not yet been evaluated in detail in inflammatory conditions.

 Our study investigated effects of salbutamol on some oxidative parameters such as MPO and SOD activities and LPO and GSH levels during the acute phase of inflammation. In inflamed tissues, activities of MPO and LPO are significantly increased. In the present case, both doses of salbutamol and indomethacin decreased the carrageenan-induced aggravation of MPO and LPO. MPO is an enzyme found primarily in azurophilic granules of neutrophils and is used as a marker for tissue neutrophil content. Its inhibition implies the presence of anti-inflammatory activity [47, 70]. Tissue MPO activity is a sensitive and specific marker for acute inflammation and reflects polymorphonuclear cell infiltration of the parenchyma. In accordance with the literature [71, 72], MPO activity in the present study significantly increased in the paw at the 4th hour after carrageenan injection when compared to healthy control rats. A variety of anti-inflammatory drugs (e.g., diclofenac, indomethacin, naproxen, piroxicam, and tenoxicam) have been shown to similarly depress the increases in myeloperoxidase activity during inflammation [73, 74].

 Lipid peroxidation has been reported to increase in inflammatory conditions [38, 75, 76]. As a marker of oxidative damage, lipid peroxidation indicates changes in membrane fluidity and permeability and thus increases in rates of protein degradation, which will eventually lead to cell lysis [77]. Increased concentrations of LPO in tissue have been reported in the carrageenan-induced inflammation model [78]. In our study, LPO content was high in carrageenan-induced inflamed paws; however, salbutamol administration prevented this increase in the LPO content of the paws.

 Tissue damage related to oxidative stress can be reversed via SOD enzyme and GSH. The action of these parameters limits the cytotoxic effects of toxic free radicals [79, 80]. In our study, salbutamol also significantly increased both GSH content and SOD activity in inflammatory paws compared to control paws. In many laboratory models and in a few clinical trials, SOD has proven to be therapeutically useful in protecting injured tissues (e.g., by ischemia, inflammation, hyperoxia, etc.) from one of these active oxygen species, the superoxide radical [80]. Preventive effects of salbutamol on superoxide generation and peroxidase release from stimulated human granulocytes [24] also supports our results. GSH has pleiotropic roles including the maintenance of cells in a reduced state, serving as an electron donor for certain antioxidative enzymes (e.g., glutathione peroxidase), and in the formation of conjugates with some harmful endogenous and xenobiotic compounds via catalysis of glutathione s-transferase [79]; thus, the ameliorating effects of salbutamol on GSH demonstrated a further beneficial effect of its administration. These results may also suggest that salbutamol attenuated the carrageenan-induced inflammation by preventing oxidative stress.

In conclusion, salbutamol, a bronchodilator agent used for asthma treatment, can effectively decrease both acute (carrageenan-induced) and chronic (cotton-pellet-induced) inflammation and propranolol reversed the anti-inflammatory effects of salbutamol. Stimulation of *β*-2 adrenergic receptors may be the underlying mechanism responsible for the observed anti-inflammatory effects. Since inflammation also takes part in asthma etiopathology, these observations may be of clinical relevance. Salbutamol also exerted significant antioxidative effects, which could at least partially explain the mechanism underlying its anti-inflammatory effects. This study may also shed light on the roles of *β*-2 adrenergic receptors in inflammatory and algesic conditions.

## Figures and Tables

**Table 1:** Effects of salbutamol and indomethacin on carrageenan-induced inflammatory paw edema in rats.

Drugs	Increase in inflammatory paw volume (mL)				Anti-inflammatory effect			
	1st h	2nd h	3rd h	4th h	1st h	2nd h	3rd h	4th h
SAL-1	0.14 ± 0.02*	0.28 ± 0.13*	0.27 ± 0.05*	0.27 ± 0.03*	43.9	43.4	44	37.5
SAL-2	0.13 ± 0.03*	0.26 ± 0.05*	0.25 ± 0.04*	0.22 ± 0.03*	44.6	48.1	48.5	48.8
IND-25	0.19 ± 0.01*	0.29 ± 0.06*	0.28 ± 0.02*	0.22 ± 0.02*	27.7	40.3	42.7	48.4
Control	0.25 ± 0.03	0.49 ± 0.06	0.49 ± 0.02	0.43 ± 0.02	—	—	—	—

SAL-1: salbutamol 1 mg/kg; SAL-2: salbutamol 2 mg/kg; IND: indomethacin 25 mg/kg. *Significant at *P* < 0.05 when compared to control. (All groups received an intraplantar injection of 0.1 mL, 1% carrageenan.)

**Table 2:** Effects of salbutamol and indomethacin on carrageenan-induced inflammatory paw nociception in rats.

Drugs	Decrease in nociceptive threshold (g)			Analgesic effect (%)		
	1st h	2nd h	3rd h	1st h	2nd h	3rd h
SAL-1	18.2 ± 1.3*	25.7 ± 2.4*	30.2 ± 3.3*	57	44.2	36.9
SAL-2	16.7 ± 2.7*	24.0 ± 4.8*	27.3 ± 4.1*	61.1	47.8	42.9
IND	12.2 ± 2.2*	20.7 ± 4.4*	19.8 ± 5.1*	71.6	55	58.5
Control	42.8 ± 5.4	46.0 ± 2.5	47.8 ± 2.7	—	—	—

SAL-1: salbutamol 1 mg/kg; SAL-2: salbutamol 2 mg/kg; IND: indomethacin 25 mg/kg. *Significant at *P* < 0.05 when compared to control. (All groups received an intraplantar injection of 0.1 mL, 1% carrageenan.)

**Table 3:** Effects of salbutamol and indomethacin cotton pellet granuloma test.

Drugs	Dose (mg/kg)	Initial weight of the cotton pellets (mg)	Wet weight of cotton pellets that were removed after 8 days (mg)	Inhibition in granuloma formation (%)
Salbutamol	1	7 ± 1	94.0 ± 4.1**	44.3
Salbutamol	2	7 ± 1	116.7 ± 3.0*	30.8
Indomethacin	5	7 ± 1	41.3 ± 3.4**	75.5
Control	—	7 ± 1	168.7 ± 4.3	—

*Significant at *P* < 0.05 when compared to control, **significant at *P* < 0.01 when compared to control.
